# Improvement of a Yairipok Chujak Maize Landrace from North Eastern Himalayan Region for β-Carotene Content through Molecular Marker-Assisted Backcross Breeding

**DOI:** 10.3390/genes12050762

**Published:** 2021-05-18

**Authors:** Maqbool Qutub, Sarankumar Chandran, Krishnakumar Rathinavel, Vellaikumar Sampathrajan, Ravikesavan Rajasekaran, Sudha Manickam, Karthikeyan Adhimoolam, Samuel Jeberson Muniyandi, Senthil Natesan

**Affiliations:** 1 Department of Plant Biotechnology, Centre for Plant Molecular Biology and Biotechnology, Tamil Nadu Agricultural University, Coimbatore 641003, India; maqbool.qutub@gmail.com (M.Q.); kumar241193@gmail.com (K.R.); sudhatamil@gmail.com (S.M.); 2 Department of Plant Molecular Biology and Bioinformatics, Centre for Plant Molecular Biology and Biotechnology, Tamil Nadu Agricultural University, Coimbatore 641003, India; saran32388@gmail.com; 3 Department of Biotechnology, Agricultural College and Research Institute, Tamil Nadu Agricultural University, Madurai 625104, India; vellai1973@yahoo.co.in (V.S.); karthik2373@gmail.com (K.A.); 4 Department of Millets, Center for Plant Breeding and Genetics, Tamil Nadu Agricultural University, Coimbatore 641003, India; chithuragul@gmail.com; 5 AICRP-MULLARP, Directorate of Research, Central Agricultural University, Imphal 795004, India; samuel8142@gmail.com

**Keywords:** CAUM66 (Yairipok Chujak), *crtRB1*, MABC, North Eastern Himalayan region

## Abstract

In the North Eastern Himalayan region (NEHR) of India, maize is an important food crop. The local people cultivate the maize landraces and consume them as food. However, these landraces are deficient in β-carotene content. Thus, we aimed to incorporate the *crtRB1* gene from UMI285β^+^ into the genetic background of the NEHR maize landrace, Yairipok Chujak (CAUM66), and thereby enhance the β-carotene content through marker-assisted backcrossing (MABC). In this regard, we backcrossed and screened BC_1_F_1_ and BC_2_F_1_ plants possessing the heterozygous allele for *crtRB1* and then screened with 106 polymorphic simple sequence repeat (SSR) markers. The plants having maximum recurrent parent genome recovery (RPGR) were selected in each generation and selfed to produce BC_2_F_2_ seeds. In the BC_2_F_2_ generation, four plants (CAUM66-54-9-12-2, CAUM66-54-9-12-11, CAUM66-54-9-12-13, and CAUM66-54-9-12-24) having homozygous *crtRB1*-favorable allele with maximum RPGR (86.74–90.16%) were selected and advanced to BC_2_F_3_. The four selected plants were selfed to produce BC_2_F_3_ and then evaluated for agronomic traits and β-carotene content. The agronomic performance of the four lines was similar (78.83–99.44%) to that of the recurrent parent, and β-carotene content (7.541–8.711 μg/g) was on par with the donor parent. Our study is the first to improve the β-carotene content in NEHR maize landrace through MABC. The newly developed lines could serve as potential resources to further develop nutrition-rich maize lines and could provide genetic stock for use in breeding programs.

## 1. Introduction

Maize is an important cereal crop grown in various agro-climatic regions and is a major source of food security and economic development in Asia, Africa, and Latin America. A total harvest area of maize is about 187 million hectares, producing 1138 million tonnes worldwide [1]. In India, maize is a major cereal crop, alongside rice and wheat. The consumption of maize increased exponentially in the last few years in both fresh and processed food markets in the urban population in India. In the North Eastern Himalayan region (NEHR), maize is the principal crop ensuring food security after rice. It is traditionally cultivated in the NEHR, particularly in Manipur, Sikkim, Arunachal Pradesh, and Tripura. The NEHR of India has been the bedrock of maize diversity with unique collections of landraces. These landraces possess valuable agronomic traits and have the potential to survive in stress conditions, therefore, they are safely preserved and used by farmers from the NEHR [2]. Moreover, the NEHR people consume green cob in different forms in their diet. People use maize to prepare traditional foods such as soup, chapati, chujak angouba (stir fry), chujak arouba (roast), chujak afutpa (boil), and chujak kabok [3]. These dishes are made mostly using landraces such as Yairipok Chujak (CAUM66), Chujak Amubi (CAUM19), Chakhao Chaujak (CAUM39), Chujak Angoubi (CAUM36), Chujak (CAUM2), and Chujak (CAUM3), which are popular among Manipuri tribes and local populations [2]. However, no prior information is available on the β-carotene content of NEHR maize landraces, which is important for its enhancement of provitamin A. Vitamin A deficiency is a major problem and causes severe health issues in children and pregnant women. The main results of vitamin A deficiency are night blindness, reduced growth, and high mortality and morbidity. The traditional habit and daily nutrient uptake of maize in the vulnerable population are key factors that prevent vitamin A deficiency.

Molecular marker-assisted backcross breeding (MABC) is reported to be a very promising breeding strategy, because it provides a fast and accurate selection of the desired genes. The use of MABC has provided a better outcome in the maize breeding programs for improving the nutritional traits [4]. So far, two major genes (*crtRB1* (β-carotene hydroxylase 1), and *LcyE* (lycopene epsilon cyclase)) have been reported to exhibit significant effects on increasing the content of β-carotene in maize [5,6]. β-carotene is hydroxylated to β-cryptoxanthin by the *crtRB1* gene and has been reported to have greater potential in the enhancement of β-carotene content than *LcyE* [7]. Yan et al. [6] identified three types of polymorphism in the *crtRB1* 3′TE gene, viz., 5;TE, InDel4, and 3;TE, which could be expressed as 3′TE allele 1(without insertion), 3′TE allele 2 (with 325 bp insertion) and 3′TE allele 3 (with 1250 bp insertion). Hence, allele 1 is identified as a favorable allele that is responsible for the accumulation of a higher level of β-carotene in the maize endosperm. Many researchers used the gene-specific markers developed from the *crtRB1* gene in MABC programs to increase the β-carotene content in popular elite varieties and hybrids [8,9,10,11]. In the NEHR of India, maize is the staple food crop and the local people cultivate the maize landraces and consume them as food. Yairipok Chujak (CAUM66), popular and widely cultivated in landrace from NEHR, is famous for its characteristics (i.e., long cob, large round seeds, and sweetness). Herein, we report our demonstration of MABC to increase the β-carotene content in Yairipok Chujak (CAUM66). To our knowledge, this is the first study to describe β-carotene improvement in the NEHR maize landrace.

## 2. Materials and Methods

### 2.1. Plant Materials and Generation of Backcross Population

Yairipok Chujak (CAUM66) from NEHR was used as the recurrent parent for the incorporation of *crtRB1* from the donor parent, UMI285β^+^, which is a high β-carotene inbred line developed by our research group [12]. The seeds of CAUM66 were obtained from Central Agricultural University, Imphal, India, and used in this study. In Kharif 2018, CAUM66 was crossed with UMI285β^+^ to generate the seeds of F_1_. The F_1_ plants were backcrossed to CAUM66 to produce BC_1_F_1_ seeds in the Rabi 2018 and then heterozygous BC_1_F_1_ plants were backcrossed with CAUM66 to produce BC_2_F_1_ seeds in the Kharif 2019. The healthy heterogeneous BC_2_F_1_ plant selected by the *crtRB1* gene-specific marker was planted in Rabi 2020 to produce the BC_2_F_2_ seeds. The healthy positive BC_2_F_2_ plants selected by gene-specific marker were selfed to produce BC_2_F_3_ seeds. The BC_2_F_3_ lines were planted in Kharif 2020 for evaluation of agronomic traits. All the field experiments were conducted from 2018 to 2020 at the Central Experimental Farm, Tamil Nadu Agricultural University, Coimbatore, India.

### 2.2. MABC Breeding Strategy

CAUM66 was crossed to UMI285β^+^ and the resulting F_1_ plants were backcrossed to CAUM66 to get BC_1_F_1_ seeds. The foreground selection for the *crtRB1* gene in the BC_1_F_1_ plants was conducted by *crtRB1* gene-specific marker. The heterozygous individual plants with the favorable allele were selected and then backcrossed to CAUM66 again to generate the seeds for the BC_2_F_1_ population. The second-round foreground selection cycle was done in the BC_2_F_2_ population to select the homozygous allele for the *crtRB1* locus. On the other hand, background selection in the BC_1_F_1_, BC_2_F_1_, and BC_2_F_2_ plants were targeted as the best plants to choose heterozygous with the *crtRB1* gene-specific marker. The background selection was done to examine the recurrent parent genome recovery (RPGR). It was performed via 210 simple sequence repeat (SSR) markers with known chromosomal positions distributing all 10 maize chromosomes. All of the SSR primer sequences used in background selection were obtained from the maize genome database (www.maizegdb.org, accessed on 9 March 2021) and synthesized by Eurofins Ltd., Bangalore, India. The results from genotype analysis were subjected to a chi-square test to study the segregation pattern of the gene of interest, for which the alleles were scored as A (recurrent parent), B (donor parent), and H (heterozygotes). The percentage recovery of the background genome was calculated using the formula determined by Sundaram et al. [13].

### 2.3. Molecular Marker Analysis

Genomic DNA was extracted from the two-week-old seedling using the modified CTAB (cetyltrimethyl ammonium bromide) method, and the quality of the extracted DNA was tested using 0.8% agarose gel and spectrophotometer (Eppendorf, Germany). Foreground selection was performed with the *crtRB1* gene-specific primer (65F: ACACCACATGGACAAGTTCG) and (62R: ACACTCTGGCCCATGAACAC, 66R: ACAGCAATACAGGGGACCAG). PCR amplification was carried out in the Eppendorf master cycle and the reaction was set for 10 µL possessing 25 ng/µL of sample DNA, 1 mM of dNTPs, 2 mM of MgCl_2_, 2 µM of both primers, and 1.5 U hf Taq polymerase. The amplification profile was comprised of initial denaturation at 94 °C for 5 min followed by 94 °C of denaturation for 30 s, annealing temperature set from the initial of 54 °C to 62 °C with the reduction of 0.5 °C per cycle, and 72 °C extension for 10 min for 19 cycles. Again, denaturation at 94 °C for 30 s, 60 °C of annealing for 30 s, an extension for 45 s at 72 °C, and a final extension at 72 °C for 10 min for 20 cycles. The amplified PCR products were run at the 3% agarose gel for 3 h with the addition of 5 µL bromophenol blue, and the resolution was documented after 3 h. For back ground selection, SSR analysis, a total of 10 µL composition of PCR reaction was performed with the components, as mentioned, and the amplification profile included initial denaturation (94 °C-7 min) followed by denaturation (94 °C-30 s), annealing (55 °C-30 s), extension (72 °C-45 s) for 35 cycles, and final extension (72 °C-7 min). A similar procedure for resolving the product and documentation was followed as described above.

### 2.4. Evaluation of Major Agronomic Traits of the Selected Lines

A total of 14 major agronomic traits were investigated in the selected lines to learn the morphological resemblance of the recurrent parent, CAUM66. Traits were days to tasseling (days), days to silking (days), plant height (cm), ear placement height (cm), tassel length (cm), number of tassel branches (No.), leaf length (cm), leaf breadth (cm), cob length (cm), cob weight (g), number of kernel rows per cob, number of kernels per row, 100 kernel weight (g), and single plant yield (g). These were recorded according to the IBPGR (International Board of Plant Genetic Resource) descriptors.

### 2.5. Characterization of the Selected Lines for β-Carotene Content

The β-carotene content was estimated in the selected lines following the previously described method [14]. The seeds from the selected lines were collected and stored in dark conditions to avoid light exposure. The whole extraction was done under yellow light to prevent the loss of the carotenoid compound. Estimation of β-carotene was done using high-performance liquid chromatography (HPLC) and was separated by C-18 column (2.5 μm, 4.6 × 150 mm) using Waters Spherisorb ODs and eluted with acetonitrile, methanol, and ethyl acetate in the ratio of 80:10:10. Carotenoids were identified with the flow rate of 1.0 mL/min by their retention time and their spectra, which were compared with the standard values. The β-carotene standard was reconstituted in acetone to five different concentrations (0.1 μg/g, 1 μg/g, 10 μg/g, 50 μg/g, 100 μg/g) [15] and was run along with the samples for comparison.

## 3. Results

### 3.1. Incorporation of β-Carotene Gene crtRB1 into the Background of CAUM66

**Figure 1:**
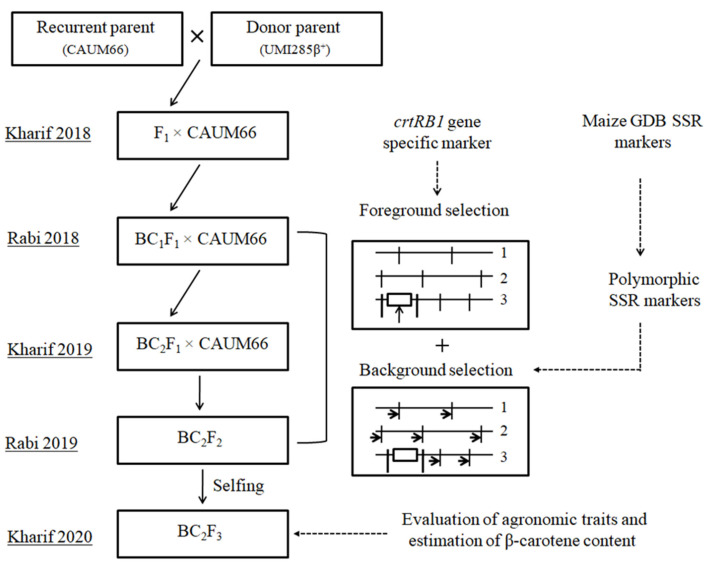
Scheme of marker-assisted backcross breeding (MABC) to improve the β-carotene content in CAUM66.

The incorporation method was carried out as detailed in Figure 1. Twenty F_1_ plants, generated via crossing between UMI285β^+^ having *crtRB1* (used as a donor parent) and the recurrent parent CAUM66, were planted and tested by the tightly linked marker to the *crtRB1* gene. The 10 healthy F_1_ plants with the positive *crtRB1* were backcrossed to produce BC_1_F_1_ seeds. The foreground selection was carried out using the *crtRB1* gene-linked marker to select the positive plants in the BC_1_F_1_ generation. A total of 124 of the 193 plants from the BC_1_F_1_ generation were found to have heterozygous *crtRB1* (Table 1). Background selection based on 106 SSR markers of polymorphism among the UMI285β^+^ and CAUM66 was done for 124 *crtRB1*-positive plants. A total of 106 polymorphic SSR markers among 210 markers distributed evenly across all the maize chromosomes were used for background analysis. Among them, 14 polymorphic markers from chromosome 10 and at least 10 polymorphic markers from the rest of the chromosomes were used to generate the data.

**Figure 2:**
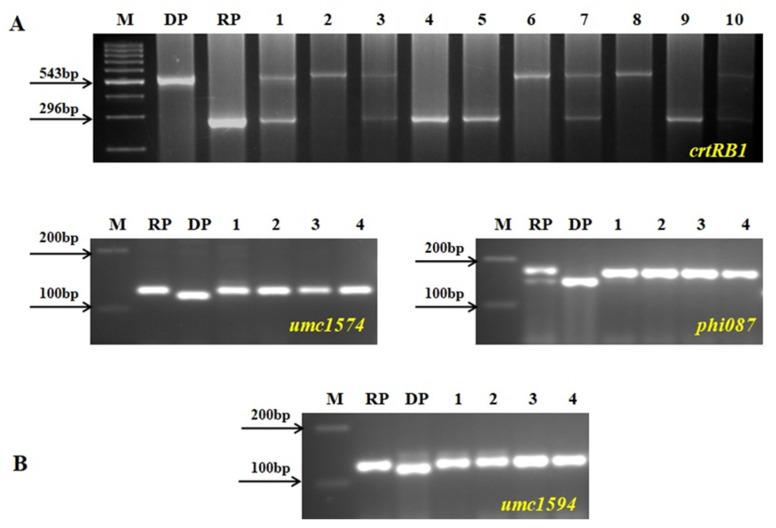
Genotyping in the backcross and selfed population of CAUM66 × UMI285β^+^. M, Ladder (100 bp); DP, donor parent; RP, recurrent parent. (**A**) Foreground screening; 1–10, segregating progenies. (**B**) Background screening: 1, CAUM66-54-9-12-2; 2, CAUM66-54-9-12-11; 3, CAUM66-54-9-12-13; 4, CAUM66-54-9-12-24.

The CAUM66-54-9 plant had the highest RPGR at 64.34%, and 63 of 106 SSR markers were restored to the background of CAUM66. Then the selected positive plant was backcrossed with CAUM66 to produce BC_2_F_1_ seeds. One hundred and fourteen *crtRB1*-positive plants were detected from the 198 BC_2_F_1_ plants. These plants were subjected to background selection using SSR markers, and RPGR ranged from 79.31% to 83.22%. The individual plant designated as CAUM66-54-9-12 had the highest RPGR of 83.22% and was selected to produce the BC_2_F_2_ population by selfing. In BC_2_F_2_ of 129 plants, 36 plants having homozygous *crtRB1* favorable allele were detected by foreground selection. Using the SSR markers, these positive plants’ background analysis revealed that 4 of 36 plants (CAUM66-54-9-12-2, CAUM66-54-9-12-11, CAUM66-54-9-12-13, and CAUM66-54-9-12-24) were found to have the maximum genome recovery of the recurrent parent (86.74–90.16%). Foreground and background results of the BC_2_F_2_ generation are presented in Figure 2. The segregation pattern of the backcross population and the background genome recovery of positive plants are presented in Table 1 and Table 2. The four selected positive plants were selfed to produce BC_2_F_3_ seeds and used for further experiments.

### 3.2. Agronomic Traits of the Selected Lines

**Figure 3:**
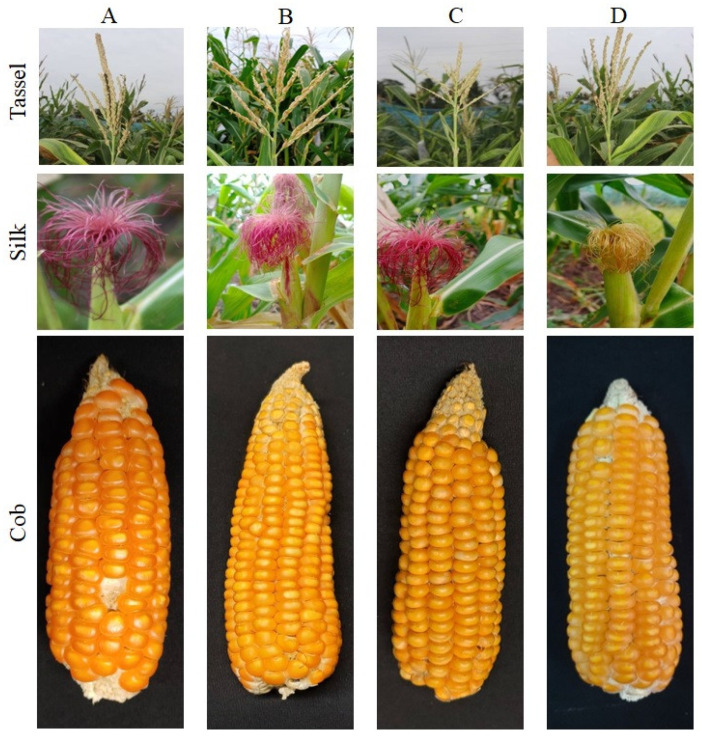
Morphological resemblance of the parents and progenies. (**A**) CAUM66; (**B**) CAUM-54-9-12-24; (**C**) CAUM-54-9-12-13; (**D**) UMI285β^+^.

Four improved lines, viz., CAUM66-54-9-12-2, CAUM66-54-9-12-11, CAUM66-54-9-12-13, and CAUM66-54-9-12-24, were obtained from the MABC scheme used to study the 14 major agronomic traits along with the recurrent parent, CAUM66. For the trait of days to tasseling, the recurrent parent recorded the mean of 59.08, and the improved lines recovery percentage ranged from 94.37% to 97.05%. CAUM66-54-9-12-13 recorded a higher recovery percentage (99.44% and 96.25%) for the plant height and cob length among the improved lines. For the leaf length, the recovery percentage ranged from 90.28% to 91.16% with an average of 90.52%. Total recovery of 94.49% and 92.91% was observed in CAUM66-54-9-12-24 for 100 kernel weight and cob weight, which was higher when compared with other improved lines. All four of the selected lines had more than 90% recovery for a single plant yield towards the recurrent parent. Finally, the line CAUM66-54-9-12-24 showed more than 90% recovery for 13 out of the 14 traits studied, excluding the number of kernel rows per cob. CAUM66-54-9-12-2 recorded more than 90% for nine traits, viz., days to tasseling, days to silking, plant height, ear placement height, tassel length, number of tassel branches, leaf length, number of kernel rows per cob and single plant yield. The improved lines’ agronomic performance is presented in Figure 3 and Table 3.

### 3.3. β-Carotene Content of the Selected Lines

β-carotene content was estimated in four improved lines, viz., CAUM66-54-9-12-2, CAUM66-54-9-12-11, CAUM66-54-9-12-13, and CAUM66-54-9-12-24. The β-carotene content value recorded 9.214 μg/g for the donor parent UMI285β^+^, and 0.86 μg/g for the recurrent parent. The improved line CAUM66-54-9-12-24 recorded the highest β-carotene (8.711 μg/g) content comparable to that of the donor parent, followed by the lines, viz., CAUM66-54-9-12-13 and CAUM66-54-9-12-11 recorded 8.647 μg/g and 8.258 μg/g, respectively. The lowest level of β-carotene was observed in the line CAUM66-54-9-12-2 with 7.541 μg/g. The improved lines’ β-carotene content is presented in Table 3.

## 4. Discussion

In the NEHR of India, maize is the staple food crop. The local people cultivate the maize landraces and consume them as food. The NEHR of India has extensive variability for the tassel, ear, and kernel characteristics in maize. These landraces possess valuable agronomic characteristics and are well-adjusted to stress conditions [16,17]. Some of the interesting trait-specific local landraces reported from this region were maintained in the maize germplasm collections of National Bureau of Plant Genetic Resources, New Delhi, India [18]. NEHR maize landraces have considerable variation in carotenoid content [19]. Recently, our research group characterized the *crtRB1* gene and explored the β-carotene content variations in the maize landraces from the NEHR of India. Yairipok Chujak (CAUM66) is a popular maize landrace in the NEHR of India, having a heterozygous allele of the *crtRB1* gene [2]. In the present study, after a season of selfing, we selected all the homozygous *crtRB1* unfavorable-allele lines from CAUM66 (data is not shown) and efficiently introduced β-carotene gene *crtRB1* into the CAUM66 by MABC, with accurate foreground selection of *crtRB1* gene-associated marker and genetic background selection with 106 polymorphic SSR markers in each generation. We obtained four plants (CAUM66-54-9-12-2, CAUM66-54-9-12-11, CAUM66-54-9-12-13, and CAUM66-54-9-12-24) with RPGR of 86.74–90.16% in the BC_2_F_2_ population. Advancement and further evaluation of these plants revealed that, apart from the considerable improvement of β-carotene content, the major agronomic traits of the four lines in BC_2_F_3_ were very similar to those of the recurrent parent, which proved the success of this MABC program.

The goal of the MABC program is to eliminate the effect of the donor parent by reducing the percentage of donor genome in order to maintain the recurrent parent characteristics in the improved lines apart from the transfer of targeted genes for the associated traits [20]. Traditional breeding methods require a minimum of six backcrossings to achieve more than 90% of RPGR ideally. However, background selection assists in achieving this RPGR percentage in BC_4_, BC_3_, or even BC_2_ [21,22]. In this study, one *crtRB1* gene-specific marker for foreground selection and 106 polymorphic SSR markers covering the entire maize genome were applied for background selection in BC_1_ and BC_2_. Additionally, individual plants having *crtRB1* allele with the maximum RPGR were selected and backcrossed with CAUM66. In BC_2_F_2_, the individual plants having homozygous *crtRB1* favorable allele were obtained by conducting the foreground and background selection, and residual fragments of donors were successfully removed.

In this study, the percentage of RPGR in four plants (CAUM66-54-9-12-2, CAUM66-54-9-12-11, CAUM66-54-9-12-13, and CAUM66-54-9-12-24) from the BC_2_F_2_ generation ranged from 86.74% to 90.16%, with line CAUM66-54-9-12-24 showing the maximum RPGR (90.16%) [23,24]. On the other hand, the evaluation of agronomic traits is helpful to determine the performance of improved lines and helps in comparing them with the recurrent parent. The recovery percentage of agronomic traits of the four improved lines had an average of 90.76%. The similarity to recurrent parent CAUM66 denotes that the improved lines and the recurrent parent (CAUM66) have significant resemblance in agronomic traits. Among these improved lines, CAUM66-54-9-12-24 expressed more than 90% recovery for 13 of the 14 traits studied, excluding the number of kernel rows per cob. For example, the agronomic traits, viz., single plant yield, days to tasseling, and days to silking, expressed more than 90% resemblance to the recurrent parent. The high degree of resemblance with their recurrent parent for the trait of plant height in the present study agreed with earlier reports of Mehta et al. [25] when developing sweet corn hybrids for vitamin A fortification. The high rate of recurrent parent recovery in the BC_2_F_2_ generation was also reported in previous studies [8,9,11,26]. The improved line CAUM66-54-9-12-24 recorded high β-carotene (8.711 μg/g) comparable to that of the donor parent (UMI285β^+^) followed by the lines, viz., CAUM66-54-9-12-13 and CAUM66-54-9-12-11 which recorded 8.647 μg/g and 8.258 μg/g, respectively. The introgression of the *crtRB1* allele from UMI285β^+^ inbred resulted in a higher accumulation of β-carotene in CAUM66-54-9-12-24 in the present study. Likewise, two to four times higher β-carotene over the original parental type was reported previously in a *crtRB1* favorable allele introgression program [8,9,27,28].

In this study, the β-carotene content of the four improved lines was on par with that of the donor parent. The lines also showed better agronomic performance, comparable to that of the recurrent parent, CAUM66. The four lines are β-carotene-rich versions, which can replace CAUM66 among the farmers from the NEHR of India. However, the lines need to be tested by multi-location trial in the various sites of the NEHR of India before use. The β-carotene-enriched Yairipok Chujak (CAUM66) will help to alleviate vitamin A deficiency in tribal areas of NEHR. This is first report to improve the β-carotene content in NEHR maize landrace through MABC. The newly developed lines could be utilized as a potential source of genetic material for improving the nutritional traits in maize breeding programs.

## Figures and Tables

**Table 1:** Segregating pattern of *crtRB1* allele in the BC_2_F_2_ population of CAUM66 × UMI285β^+^.

S. No.	Generation	No. of Plants Screened	Favorable Allele (1)	Heterozygotes (1 and 3)	Unfavorable Allele (3)	Chi-Square Value	*p*-Value
1.	BC_1_F_1_	193	0	124	69	15.673	0.000075 **
2.	BC_2_F_1_	198	0	114	84	4.545	0.033006 ^ns^
3.	BC_2_F_2_	129	36	64	29	0.7674	0.681321 ^ns^

Note: ** Highly significant (distorting from the Mendelian ratio); ^ns^ Non-significant (no distortion from the normal Mendelian ratio).

**Table 2:** Details of the recurrent parent genome recovery (RPGR) in the foreground positive plants.

S. No.	Generations	Progenies Selected	RPG (%)	No. of Markers Restored	Range of RPG (%) among the Selected Progenies
1.	BC_1_F_1_	CAUM66-2-27	63.75	59–63	61.82–64.34
		CAUM66-28-35	62.96		
		CAUM66-15-12	61.82		
		CAUM66-54-9	64.34		
2.	BC_2_F_1_	CAUM66-54-9-7	82.12	78–84	79.31–83.22
		CAUM66-54-9-11	81.01		
		CAUM66-54-9-12	83.22		
		CAUM66-54-9-18	79.31		
3.	BC_2_F_2_	CAUM66-54-9-12-2	86.74	83–94	86.74–90.16
		CAUM66-54-9-12-11	87.71		
		CAUM66-54-9-12-13	88.86		
		CAUM66-54-9-12-24	90.16		

**Table 3:** Agronomic performance and β-carotene content of the four improved lines in BC_2_F_3_ generation.

S. No.	Traits	Recurrent Parent	Donor Parent	CAUM66-54-9-12-2		CAUM66-54-9-12-11		CAUM66-54-9-12-13		CAUM66-54-9-12-24	
		Mean	Mean	Mean	RGP%	Mean	RGP %	Mean	RGP %	Mean	RGP %
1	Days to tasselling	59.08	57.64	57.24	96.88	56.47	95.58	55.73	94.37	57.34	97.05
2	Days to silking	61.04	61.01	60.21	98.64	59.84	98.03	58.35	95.59	59.57	97.59
3	Plant height	168.82	120.60	157.48	93.28	143.25	84.85	167.89	99.44	161.45	95.63
4	Ear placement height	69.78	58.60	65.47	93.82	63.47	90.95	64.25	92.07	65.48	93.83
5	Tassel length	36.80	19.02	33.29	90.46	34.25	93.07	31.27	84.97	35.87	97.47
6	Number of tassel branches	08.04	20.32	07.58	94.27	07.54	93.78	07.58	94.27	07.53	93.65
7	Leaf length	60.09	56.32	54.25	90.28	54.27	90.31	54.28	90.33	54.78	91.16
8	Leaf breadth	07.41	06.31	06.28	84.75	06.59	88.93	06.57	88.66	06.87	92.71
9	Cob length	16.29	13.16	14.65	89.93	13.59	83.42	15.68	96.25	15.41	94.59
10	Number of kernel rows per cob	11.12	10.58	10.74	96.58	10.14	91.18	9.25	83.18	9.57	86.06
11	Number of kernels per row	19.84	26.62	17.69	89.16	16.25	81.90	15.64	78.83	18.21	91.78
12	100 kernel weight	25.78	23.85	21.45	83.20	22.87	88.71	21.87	84.83	24.36	94.49
13	Cob weight	95.51	74.93	80.64	84.43	80.54	84.32	85.49	89.50	88.74	92.91
14	Single plant yield	66.57	62.31	64.33	96.63	62.34	93.64	65.34	98.15	63.58	95.50
15	Beta carotene content (µg/g)	0.86	9.214	7.541		8.258		8.647		8.711	

## Data Availability

The datasets generated or analyzed during the current study are available from the corresponding author on reasonable request.
